# Long-term Outcomes of Feminizing Genitoplasty in DSD: Genital Morphology, Sensitivity, Sexual Function, and Satisfaction

**DOI:** 10.1210/jendso/bvaf014

**Published:** 2025-02-19

**Authors:** Min Jeong Bag, Marlene Inacio, Tânia Aparecida Sartori Sanchez Bachega, Rafael Loch Batista, Guiomar Madureira, Elaine Maria Frade Costa, Sorahia Domenice, Berenice Bilharinho Mendonca, Francisco Tibor Dénes, Maria Helena Palma Sircili

**Affiliations:** Uropediatric Unit, Division of Urology, Hospital das Clínicas, Faculdade de Medicina, Universidade de São Paulo, São Paulo 05403-900, Brazil; Pediatric Surgery Unit, Division of Surgery, Escuela de Medicina, Facultad de Medicina, Pontificia Universidad Catolica de Chile, Santiago 8331150, Chile; Developmental Endocrinology, Division of Endocrinology, LIM/42, Hospital das Clinicas, Faculdade de Medicina, Universidade de São Paulo, São Paulo 05403-900, Brazil; Developmental Endocrinology, Division of Endocrinology, LIM/42, Hospital das Clinicas, Faculdade de Medicina, Universidade de São Paulo, São Paulo 05403-900, Brazil; Developmental Endocrinology, Division of Endocrinology, LIM/42, Hospital das Clinicas, Faculdade de Medicina, Universidade de São Paulo, São Paulo 05403-900, Brazil; Developmental Endocrinology, Division of Endocrinology, LIM/42, Hospital das Clinicas, Faculdade de Medicina, Universidade de São Paulo, São Paulo 05403-900, Brazil; Developmental Endocrinology, Division of Endocrinology, LIM/42, Hospital das Clinicas, Faculdade de Medicina, Universidade de São Paulo, São Paulo 05403-900, Brazil; Developmental Endocrinology, Division of Endocrinology, LIM/42, Hospital das Clinicas, Faculdade de Medicina, Universidade de São Paulo, São Paulo 05403-900, Brazil; Developmental Endocrinology, Division of Endocrinology, LIM/42, Hospital das Clinicas, Faculdade de Medicina, Universidade de São Paulo, São Paulo 05403-900, Brazil; Uropediatric Unit, Division of Urology, Hospital das Clínicas, Faculdade de Medicina, Universidade de São Paulo, São Paulo 05403-900, Brazil; Uropediatric Unit, Division of Urology, Hospital das Clínicas, Faculdade de Medicina, Universidade de São Paulo, São Paulo 05403-900, Brazil

**Keywords:** differences of sex development, congenital adrenal hyperplasia, genitalia, female, sensory thresholds, sexual dysfunction

## Abstract

**Context:**

Understanding long-term outcomes and patient satisfaction with feminizing genitoplasty (FG) in patients with differences of sexual development (DSD) is crucial for optimizing treatment protocols.

**Objective:**

To evaluate long-term morphological and functional results and patients’ satisfaction in a cohort of DSD patients submitted to FG.

**Design:**

Cross-sectional and retrospective cohort study conducted from 1965 to 2016 with follow-up assessments.

**Setting:**

Tertiary care center.

**Patients or Other Participants:**

Sixty DSD female patients, including 36 with congenital adrenal hyperplasia (CAH) and 24 with non-CAH DSD etiology, who underwent FG.

**Intervention(s):**

FG procedures were performed, and results were analyzed based on age at surgery and surgical techniques used.

**Main Outcome Measure(s):**

Surgical results, genital sensitivity, sexual function, and patient satisfaction.

**Results:**

Ninety-one percent of patients had normal clitoral size, and 85% had separated perineal orifices. Three patients with persistent urogenital sinus did not report symptoms. Genital sensitivity to mechanical and vibratory stimuli was similar to control groups. No CAH patients experienced overall sexual dysfunction, while 3 non-CAH patients and 4 control women reported reduced sexual desire and arousal. Eighty-nine percent of patients preferred surgery during childhood, and 97% were satisfied with their surgical outcomes.

**Conclusion:**

FG outcomes in this cohort were satisfactory, with no significant impact on genital sensitivity or sexual function. Most patients preferred early surgery and reported high satisfaction with the results. Further studies are needed to confirm these findings in broader populations.

The ideal approach for patients with differences of sexual development (DSD) and atypical genitalia is being widely discussed, particularly concerning their surgical management [1]. Surgical techniques of feminizing genitoplasty (FG) have evolved over the decades, leading to fewer complications. However, controversies still exist regarding the ideal age to perform the operation, long-term complications, and the need for reoperation [2].

Concerns about genital sensitivity, long-term complications, and the need for reoperations in adulthood led some authors to review the surgical intervention and discuss the ideal age to perform surgery [1].

Over the years, the management of DSD patients improved after a better understanding of the genital anatomy, the development of diagnostic methods, and the creation of specialized centers with a multidisciplinary team, including psychological support until adulthood. Surgeons created new techniques to improve the preservation of genital sensitivity and also promote better cosmetic appearance and function of the female genitalia [2]. Studies of long-term patient evaluation until adulthood brought a better understanding of the patient's functional problems and sexual behavior in the different diagnoses of DSD with genital atypia.

However, there are very few publications that discuss patient satisfaction and functional results using different evaluation methods, which leads to misconceptions about surgical outcomes [3, 4].

This study aimed to evaluate the long-term results of a cohort of female DSD patients submitted to FG over a period of 50 years, using different evolving techniques.

## Patients

We evaluated 60 patients submitted to FG and treated by an interdisciplinary team in a single tertiary center from 1965 until 2016. Thirty-six were 46,XX DSD due to congenital adrenal hyperplasia (CAH), 3 46,XX DSD of unknown etiology, 16 females with 46,XY DSD secondary to several etiologies, and 5 chromosomal DSD (Table 1).

**Table 1.** DSD etiology

DSD etiology	n
DSD 46,XX	
CAH	36
Undetermined	3
DSD 46,XY	
Gonadal dysgenesis	2
17-β-HSD III deficiency	5
5-α-reductase II deficiency	3
Partial androgen insensitivity syndrome	5
Undetermined	1
Chromosomal DSD	
Gonadal dysgenesis	4
DSD ovotesticular 46,XX/46,XY	1

Abbreviations: CAH, congenital adrenal hyperplasia; DSD, differences in sexual development; HSD, hydroxysteroid dehydrogenase.

## Methods

This is a retrospective and cross-sectional study approved by our institutional ethical committee (no. 1.645.341). Seventy patients were invited for this study, and 60 agreed to participate. All the patients and/or parents signed an informed consent. Clinical records were reviewed to retrieve perioperative data including anatomical findings before surgery, surgical descriptions, complications, and reoperations. The cross-sectional part of the study involved physical examinations, sensitivity tests, semistructured interviews, and the validated Female Sexual Function Index (FSFI) to assess sexual function.

A surgical technique for treating the urogenital sinus (UGS) with a cut-back incision was used in 1 patient with CAH (Prader II) and in 3 patients with other etiologies of DSD (2 Prader II and 1 Prader III); however, this technique was abandoned after 1990.

The Y-V perineal flap to treat the UGS has been employed since 1990. After 2013, to better expose both orifices in the perineum (urethra and vaginal introitus), the modified partial mobilization of the UGS was associated with the Y-V perineal flap; in this technique, the posterior wall of the UGS is dissected to bring the vaginal introitus to the perineal surface.

Concerning clitoromegaly surgical approaches, clitoridectomy was performed in only 2 patients in 1970 and 1988, and it was abandoned after this period. The clitoroplasty without preservation of the dorsal neurovascular bundle was used until 1998. Since 1998, all patients who submitted to clitoroplasty had preservation of the neurovascular bundle.

At the current follow-up, patients submitted to a genital examination and sensitivity tests by the same researcher and answered questionnaires assessing sexual function and satisfaction with surgery. Four patients only answered questionnaires, declining physical examination.

For statistical analyses, we divided our patients into 2 groups according to their DSD etiology in CAH and non-CAH patients.

A morphological evaluation consisted of a genital examination with measurement of clitoral size and identification of perineal orifices. The vaginal length was evaluated by digital examination in patients who already had sexual intercourse.

Regarding genital sensitivity, the clitoris or clitoral area and the lower vagina (vulvar vestibule) were evaluated by applying graduated von Frey filaments (SENSELab Aesthesiometer™, SOMEDIC, Sweden) to assess light touch and mechanical discomfort thresholds. Patients also scored the degree of discomfort with a visual analog scale. Allodynia (pain due to a stimulus that does not normally provoke pain) and vibratory sensitivity were assessed in the same genital areas by applying the tip of a tissue towel and a vibratory device (VibraTip™, University Hospitals Bristol NHS Foundation Trust, UK), respectively.

Patients who were sexually active in the last month answered the validated Portuguese version of the FSFI [5], which assesses 6 domains: desire, arousal, lubrication, orgasm, satisfaction, and pain. An FSFI score of less than 26.5 is considered abnormal [6].

Regarding satisfaction, patients and parents answered a questionnaire using a 5-point Likert scale (1 = strongly disagree; 5 = strongly agree) regarding their satisfaction with FG and the ideal time for surgery.

The control group consisted of mothers of our pediatric patients who participated in the study. The selection criteria were based on their age, sexual activity, and genital surgery. Fifteen sexually active adult women without previous genital surgery were recruited for evaluation of genital sensitivity and sexual function.

We performed statistical analysis with Stata/SE 12.0 for Mac. We analyzed the association between surgical results and age at surgery by the Mann-Whitney test and surgical results and surgical techniques by the Fisher exact test. The Mann-Whitney test assessed associations between morphological and sensorial findings. Sensorial findings and sexual function questionnaire results were compared among the groups by the Kruskal-Wallis test, and the correlation between these results was analyzed by Spearman rank-order correlation. A *P*-value equal to or less than .05 was considered statistically significant.

## Results

### Clinical Records Review

The genital grade of virilization at diagnosis was Prader III and IV in more than 70% of CAH patients; while in non-CAH patients, lower scores (Prader II and III) were more frequent. This difference in the grade of virilization between the groups was statistically significant (*P* = .03) (Table 2).

**Table 2.** Preoperative Prader score

Etiology	Prader score				
	I	II	III	IV	V
CAH (%)	3	17	34	40	6
non-CAH (%)	4.5	46	36	9	4.5

Abbreviation: CAH, congenital adrenal hyperplasia.

The median chronological age at surgery of CAH patients was 2 years (1.2 to 33 years) and of non-CAH patients was 9.5 years (1 to 34 years). Forty-eight patients underwent 1-staged clitoral and UGS repair, 11 patients had only clitoral surgery, and 1 patient had only UGS repair. Surgical techniques are detailed in Table 3.

**Table 3.** Surgical techniques for clitoris reduction and UGS repair

Clitoris reduction	CAH	Non-CAH
Clitoridectomy (1970 and 1988)	1	1
Clitoroplasty without DNVB preservation (until 1998)	14	12
Clitoroplasty with DNVB preservation	21	10

UGS repair	CAH	non-CAH
Cut-back (until 1990)	1	3
Y-V perineal flap without vaginal mobilization	16	11
Y-V perineal flap with posterior vaginal mobilization (since 2013)	14	4

Abbreviations: CAH, congenital adrenal hyperplasia; DNVB, dorsal neurovascular bundle; UGS, urogenital sinus.

Forty-eight patients (80%) had good surgical results and did not need further interventions. However, 5 CAH and 7 non-CAH patients presented complications (Table 4). Early complications consisted of hematomas, wound infection, and granulomas, while late complications were symptomatic persistence of UGS, vaginal stenosis, and clitoromegaly.

**Table 4.** Number of intraoperative and postoperative surgical complications

Complication	CAH	Non-CAH
Recurrent urinary tract infection*^a^*	3	1
Vaginal introitus stenosis	1	1
Hematocolpos	1	0
Urethral lesion (intraoperative)	0	1
Clitoromegaly (de novo)	1	1
Granuloma	1	3
Hematoma	0	1
Wound infection	0	1

Abbreviation: CAH, congenital adrenal hyperplasia.

^*a*^Due to urogenital sinus persistence.

Three patients had more than 1 complication: 1 CAH patient with persistent UGS had vaginal introitus stenosis and hematocolpos requiring 2 reinterventions; 1 non-CAH patient presented an intraoperative urethral lesion and a vaginal introitus stenosis 12 years after surgery; and another non-CAH patient had wound infection and granuloma. No patient had significant bleeding or wound dehiscence. There was no statistically significant association between complication proportion and age at surgery (*P* = .14) or surgical technique.

Three patients needed reoperations during the early postoperative period, and 8 had reintervention after 6 months of FG (Table 5).

**Table 5.** Reoperation and time between interventions

Reoperation	CAH (n = 5)	non-CAH (n = 6)	Median of years after the first surgery (range)
Granuloma excision	1	3	0.6 (0.25-2)
Redo UGS repair	3	1	7 (0.5-11)
Enlargement of vaginal introitus	1*^a^*	1	10.5 (9-12)
Redo clitoroplasty	1	1	4.5 (4-5)

Abbreviations: CAH, congenital adrenal hyperplasia; UGS, urogenital sinus.

^*a*^Previously submitted to redo of UGS repair.

### Current Follow-up

The median chronological age of our patients at examination was 25 years (3 to 55 years) and the median follow-up was 12 years (ranging from 1 to 52 years).

#### Morphological results

A clitoris was present in 91% of examined patients of each group (Table 6).

**Table 6.** Clitoral reduction techniques and outcome

Clitoral reduction techniques	CAH (n = 33)		Non-CAH (n = 22)	
	Present clitoris	Absent clitoris	Present clitoris	Absent clitoris
Clitoridectomy	0	1	0	1
Clitoroplasty without preservation of DNVB	10	2	10	1
Clitoroplasty with preservation of DNVB	20	0	10	0

Abbreviations: CAH, congenital adrenal hyperplasia; DNVB, dorsal neurovascular bundle; UGS, urogenital sinus.

The presence of the clitoris was significantly higher when the dorsal neurovascular bundle was preserved during the clitoroplasty (*P* = .04). No patient had clitoromegaly at the present follow-up. Age at surgery did not influence the outcome of clitoral surgery (*P* = .22).

Two separate perineal orifices were evidenced in 83% of CAH patients and 88% of non-CAH patients who underwent UGS repair (Table 7).

**Table 7.** Surgical technique for UGS repair and outcome

UGS surgical technique	CAH (n = 29)		Non-CAH (n = 17)	
	2 perineal orifices	Persistent UGS	2 perineal orifices	Persistent UGS
Cut-back	1	0	3	0
Perineal flap without posterior vaginal wall mobilization	10	4	8	2
Perineal flap with posterior vaginal wall mobilization	13	1	4	0

Abbreviations: CAH, congenital adrenal hyperplasia; DNVB, dorsal neurovascular bundle; UGS, urogenital sinus.

Five percent of the patients who were operated on using the perineal flap with mobilization had persistent UGS, while 25% of the patients who were operated on using the perineal flap without vaginal mobilization had UGS persistence. However, this was not statistically significant (*P* = .36). Age at surgery did not influence the outcomes of UGS repair in either of the groups (*P* = .34 in the CAH group and *P* = .06 in the non-CAH group).

Seven patients had UGS persistence. Three of them had initiated sexual activity. One complained of urinary infections at initiation of sexual activity, but these ceased within a year, while the other patients were asymptomatic.

Vaginal length was measured by digital examination in 21 patients (6 patients refused this part of the examination). All 7 CAH patients had more than 7 cm of vaginal length, even in patients who did not have recent sexual activity. Ten non-CAH patients had more than 7 cm of vaginal length; 1 had 6 cm; and, 3 patients, sexually inactive for several months, had vaginal lengths shorter than 6 cm.

Genital sensitivity tests were performed in 15 CAH and 14 non-CAH patients, besides the control group. The clitoral sensitivity test showed similar light touch detection thresholds in patients with CAH and the control group. The clitoral sensitivity test in the non-CAH group showed impaired detection of light touch compared to the control group (*P* = .03). The clitoral mechanical discomfort threshold and the degree of discomfort were not statistically different between groups of patients and the control group (Table 8). Three patients without evident clitoris had sensitivity thresholds comparable to the rest of the CAH patients and control group in the clitoral area.

**Table 8.** Mechanical sensitivity test performed in the clitoris and lower vagina (vulvar vestibule)

Mechanical stimuli test	CAH (n = 15)	non-CAH (n = 14)	Control (n = 14)	*P*-value*^a^*
	Median (range)	Median (range)	Median (range)	
Clitoral				
Detection threshold (gf)	0.034 (0.034-0.145)	0.085 (0.026-0.145)	0.03 (0.026-0.145)	.03
Discomfort threshold (gf)	5.1 (1.1-110)	3.3 (0.39-50)	5.1 (0.145-34)	.92
Degree of discomfort VAS	1 (0-6.5)	1 (0-2.5)	1.25 (0-2.8)	.65
Lower vagina (vulvar vestibule)				
Detection threshold (gf)	0.085 (0.034-0.32)	0.085 (0.026-0.32)	0.0745 (0.026-0.32)	.85
Discomfort threshold (gf)	6.7 (1.7-110)	5.1 (0.39-110)	4.2 (0.145-50)	.55
Degree of discomfort VAS	1 (0-8.5)	1.75 (0-5.5)	1.5 (0-5.5)	.73

Abbreviations: CAH, congenital adrenal hyperplasia; VAS, visual analog scale.

^*a*^Kruskal-Wallis test.

The nonevident clitorises in these 3 cases were covered by skin beneath the labia minora.

Sensitivity evaluation of the lower vagina showed no statistically significant differences in the detection threshold, discomfort threshold, or degree of discomfort for mechanical stimuli between groups of patients and the control group (Table 8).

None of the examined women had allodynia (pain due to a stimulus that does not normally provoke pain) in the evaluated areas, and all of them could correctly detect the vibratory stimuli.

Results of the sensitivity evaluation did not show any significant difference when analyzed concerning the age at surgery, surgical techniques of clitoris reduction, and UGS repair.

Regarding sexual activity, 27 women had initiated sexual activity; the median age of first intercourse among CAH patients was 19.6 years, ranging from 14 to 27 years. For patients with other DSD etiologies, the median age of first intercourse was 22.06 years, ranging from 15 to 35 years.

The frequency of sexual intercourse was variable, but 13 patients reported sexual intercourse in the last 4 weeks.

Although 27 patients had initiated sexual activity, 13 patients (6 CAH and 7 non-CAH patients) and 11 control women met the criterion to answer the FSFI questionnaire. The total FSFI score of the 3 groups was similar and above 26.5 in all of them (Table 9). There were no statistically significant differences in the scores of each specific domain evaluated by the questionnaire among the groups.

**Table 9.** Female sexual function index questionnaire results in patients and control groups

Domain	46,XX CAH (n = 7)	non-CAH (n = 6)	Control group (n = 11)
Desire (1, 2-6)	3.6 (3-5.4)	3.6 (1.2-4.8)	3.6 (3-5.4)
Excitation (0-6)	4.8 (3.3-5.1)	4.65 (1.5-5.1)	4.8 (3.9-5.7)
Lubrication (0-6)	5.4 (4.8-6)	4.65 (2.7-6)	4.8 (3.3-6)
Orgasm (0-6)	4.8 (2.4-5.6)	3.8 (2.7-6)	5.6 (3.2-6)
Satisfaction (0-6)	5.2 (4.8-6)	4.8 (2.4-6)	4.8 (2.4-5.6)
Pain (0-6)	6 (4.4-6)	6 (1.6-6)	5.2 (3.6-6)
Total (2-36)	29.9 (27.8-30.2)	27.75 (11.8-32.7)	28.8 (23.1-34.5)

Abbreviation: CAH, congenital adrenal hyperplasia.

Two patients with a persistent UGS were able to have intercourse through the UGS, which was widely opened in the perineum after dilation with acrylic molds. These 2 patients had FSFI scores of 29 and 30.2, respectively, indicating good sexual function.

When individually analyzed, 3 non-CAH patients and 4 control women had scores suggestive of sexual dysfunction. More than half of these women (57%) were older than 40 years. Two patients reported low scores in desire, arousal, and lubrication but no pain at penetration. The oldest patient had the lowest total score in all the domains. None of the CAH patients had scores suggestive of sexual dysfunction.

Women with low detection thresholds at the lower vagina had better scores in desire, arousal, and satisfaction subscales and overall score (desire vs detection: Spearman's coefficient −0.47, *P* = .03; arousal vs detection: Spearman's coefficient −0.62, *P* = .003; satisfaction vs detection: Spearman's coefficient-0.5, *P* = .01 and overall sexual function score vs detection threshold: Spearman's coefficient −0.54, *P* = .01). Furthermore, the higher the grade of discomfort caused by mechanical stimuli at the lower vagina, the lower the score of lubrication (lubrication vs grade of discomfort: Spearman's coefficient −0.47, *P* = .04). There were no statistically significant associations between the results of the clitoral sensitivity test and FSFI scores.

Satisfaction with the surgical results was high among adolescent and adult CAH patients, and they favored surgery performed in childhood (Table 10). The median chronological age at surgery of this group was 2 years (ranging from 1.5 to 33 years), and the median age at current evaluation was 26.5 years (ranging from 15 to 62 years).

**Table 10.** Results of satisfaction questionnaires answered by adolescent and adult CAH and non-CAH patients and parents

	CAH % (n = 20)	Non-CAH % (n = 17)	Parents % (n = 27)
1. You are satisfied with the results of the surgery in the genitalia			
Strongly Agree	90	58	93
Agree	10	18	7
Indifferent	—	24	—
Disagree	—	—	—
Strongly disagree	—	—	—
2. It is good to perform the genitoplasty in childhood			
Strongly Agree	90	76	100
Agree	10	18	—
Indifferent	—	—	—
Disagree	—	—	—
Strongly disagree	—	6	—
3. It is good to perform the genitoplasty in adulthood			
Strongly Agree	5	12	—
Agree	—	6	—
Indifferent	10	12	3
Disagree	20	12	30
Strongly disagree	65	58	67

Abbreviation: CAH, congenital adrenal hyperplasia.

Adolescent and adult non-CAH patients were satisfied with the results of the FG. Their median age at surgery was 14 years (1 to 34 years), while age at evaluation was 30 years (15 to 55 years). The 2 patients who strongly agreed with surgery in adulthood were operated at 15 and 24 years of age, but 1 of them also strongly agreed with surgery in childhood (Table 10).

The satisfaction of parents of younger patients (20 CAH and 7 non-CAH) was also high, and they preferred FG performed during childhood (Table 10).

## Discussion

This study complements a previous evaluation of anatomical and functional outcomes of DSD patients submitted to FG performed in our institution [4]. The data presented here result from more than 50 years of experience with FG. Additionally, this is one of the few studies that has evaluated genital sensitivity with an objective method and patients’ satisfaction with the surgical results.

Our overall complication proportion was lower than previously reported [7-10] and consistent with recent publications [11-15]. Furthermore, it was not associated with the age at surgery, as some authors previously hypothesized [7, 16].

Vaginal stenosis is described in up to 82% of CAH patients submitted to FG [16]. Alizai et al reported vaginal stenosis in 13 pubertal CAH patients submitted to FG early in childhood [7]. In our study, 2 patients (3.3%) required reoperation due to vaginal stenosis: 1 was operated on with the cut-back technique and the other patient, with high confluent vagina, was operated on using the perineal flap technique without vaginal mobilization. We believe that insufficient skin flap and the anatomy of vaginas with high confluence can lead to stenosis, as already suggested by Eroglu [11] and other authors [8, 9, 14].

Persistent UGS is reported in up to 46% of patients [7]. In our previous report, 11 out of 34 CAH patients presented this condition [17]. In the current study, 4 patients were reoperated due to recurrent urinary tract infections secondary to persistent UGS. Seven patients (15%) presented UGS persistence with no symptoms. We agree with other authors that current techniques for vaginoplasty present lower proportions of complications [12, 14, 18]. Although statistically not significant, we observed less persistence of UGS when vaginal mobilization was performed.

The treatment of the UGS using a perineal flap and mobilization of the posterior wall of the UGS began at our center in 2013. Prior to that, a perineal flap without mobilization and a cut-back incision were used to open the UGS. However, the older techniques were not successful in bringing both orifices (urethra and vagina) to the surface of the perineum in patients with a long UGS (greater than 3 cm) and high vaginal confluence, leading to the persistence of the UGS. The technique involving mobilization of the posterior wall of the UGS in patients with a long UGS resulted in separated orifices on the perineum surface in all but 1 of our cases.

Techniques for clitoroplasty have improved due to better knowledge of clitoral anatomy [19, 20]. Our findings are consistent with previous studies that reported higher proportions of clitoral preservation when the dorsal neurovascular bundle is spared [7, 14, 19, 20]. This has been the standard procedure for clitoral reduction in our institution since 1998.

Genital sensitivity is an emerging field, and normative values for various tests have been published over the past decade [21]. However, the mechanisms of erogenous sensitivity remain poorly understood. Studies on genital sensitivity following FG are limited, and clear evidence suggesting a negative impact of the surgery is lacking [22]. Nevertheless, there is no concrete understanding of erogenous sensitivity mechanisms, and consequently, little is known about the relationship between genital sensitivity and sexual function [23].

In our study, unlike previous ones, we assessed thresholds for mechanical and vibratory stimuli. Theoretically, large nerve fibers, which transmit sensations of touch and vibration, should play a more important role in sexual function than small nerve fibers, which transmit sensations of temperature and pain.

There are few reports of quantitative evaluation of genital sensitivity in patients submitted to FG. Crouch et al reported impaired genital sensitivity in operated CAH patients, compared to a control group, based on differences in clitoral thermal thresholds but did not find differences in vibratory thresholds [24]. In the study of Zhen et al, 46,XX DSD patients presented decreased clitoral thermal and vibratory sensitivity compared to a control group, which was not statistically significant [21]. It is expected that large sensory fibers (involved in sensations of touch, vibration, and light pressure) should be most relevant for female sexual function, while thermal senses, served by small nerve fibers, should be less important [23]. Therefore we preferred to evaluate mechanical and vibratory sensitivity. Similarly to Zhen, we did not find differences in the clitoral sensitivity of CAH patients compared to the control group. Interestingly, 3 of the patients with nonidentified clitoris had preserved sensitivity in the clitoral site. Additionally, the impaired clitoral light touch detection of non-CAH patients did not show any association with sexual function. Like Crouch [24] and Zhen [21], we did not find differences in the lower vaginal sensitivity of our subjects compared to the control group. Furthermore, we found that lower vaginal sensitivity is associated with sexual function, while this association was not observed with clitoral sensitivity. Nonetheless, there is no clarity about the association between genital sensitivity and sexual function.

A review by Schober et al [4] concluded that sexual function is impaired in DSD patients. Crouch [24] reported a tendency of patients with the most impaired sensitivity to show worse sexual function scores. Nevertheless, nonoperated CAH patients had similar scores of global sexual dysfunction and sexual avoidance compared to operated CAH patients [24]. Our results show that DSD women have sexual function comparable to the control group, similar to the studies of Zhen [21] and Fagerholm [25]. Interestingly, even without evident clitoris and with persistent UGS, our patients had good sexual function with no complaints about orgasm or pain. Moreover, sexual dysfunction is described in up to 50% of the female population, and it is associated with aging [23].

Female patients with CAH have low fertility rates. Several factors are considered responsible, with the status of the introitus playing an important role in fertility due to limitations in sexual intercourse [26]. In our data, 4 CAH patients attempted to conceive: 2 of them had 2 children each, 1 had 1 child, and 1 experienced a spontaneous abortion early in pregnancy. All pregnancies were carried to term without complications, and the deliveries were by cesarean section. All offspring were born with typical female genitalia.

Among the patients with a persistent urogenital sinus, 2 were able to have sexual intercourse after the UGS was dilated using acrylic molds. One of these patients conceived but had a spontaneous abortion. The third patient did not express interest in sexual activity and continued to have a persistent UGS.

In our study, all parents and almost all patients preferred performing FG during childhood, and most of them were against surgery in adulthood. These results are in agreement with those reported by Wisniewski [1], Nordensköld [13], and Nokoff [27]. Furthermore, our non-CAH patients preferred to be operated on before adulthood, as did DSD 46,XY individuals evaluated by Meyer-Bahlburg et al [28]. Similarly, in the study of Fagerholm et al, DSD patients did not regret having been operated on early in life, and 13% of them felt that their surgery could have been performed even earlier [25].

There are few publications about DSD patients’ satisfaction with the surgical results of FG [29]. In our study, all parents were satisfied with the results, while no patient reported dissatisfaction. Moreover, patients who had genital surgery in childhood declared that they were relieved they did not have to grow up with atypical genitalia.

### Limitations of This Study

This study includes both retrospective and cross-sectional surgical data, covering the different techniques used in earlier years as well as more recent advanced techniques. Long-term surgical outcomes are crucial for enabling surgeons to make technical modifications aimed at improving results. Significant progress in clinical and psychological evaluation and diagnosis has also enhanced the treatment of DSD. There is no one-size-fits-all approach for all individuals; instead, an individualized approach is required, taking into account age, diagnosis, psychological assessment, and personal desires. Psychological support is essential for evaluating each individual, beginning before diagnosis and surgical procedures and continuing after surgery.

## Conclusions

We present more than 50 years of experience with FG and the evolution of surgical techniques with improved outcomes over the years. Additionally, our results show that age at surgery had no association with surgical outcomes or complications. FG did not affect overall genital sensitivity. Sexual function in active patients was comparable to the control group. Satisfaction with surgical results was high among patients and parents, and most of them favored FG performed in childhood.

## Data Availability

The data generated and analyzed during this study can be made available upon reasonable request. Researchers interested in accessing the data may contact the corresponding author, subject to applicable ethical and institutional guidelines.
